# Application of Remote Sensing for Phenotyping Tar Spot Complex Resistance in Maize

**DOI:** 10.3389/fpls.2019.00552

**Published:** 2019-04-30

**Authors:** Alexander Loladze, Francelino Augusto Rodrigues, Fernando Toledo, Felix San Vicente, Bruno Gérard, Maruthi Prasanna Boddupalli

**Affiliations:** ^1^International Maize and Wheat Improvement Center (CIMMYT), Texcoco, Mexico; ^2^International Maize and Wheat Improvement Center (CIMMYT), Kenya World Agroforestry Centre (ICRAF), Nairobi, Kenya

**Keywords:** corn, disease control, plant pathogen, new diseases, UAV

## Abstract

Tar spot complex (TSC), caused by at least two fungal pathogens, *Phyllachora maydis* and *Monographella maydis*, is one of the major foliar diseases of maize in Central and South America. *P. maydis* was also detected in the United States of America in 2015 and since then the pathogen has spread in the maize growing regions of the country. Although remote sensing (RS) techniques are increasingly being used for plant phenotyping, they have not been applied to phenotyping TSC resistance in maize. In this study, several multispectral vegetation indices (VIs) and thermal imaging of maize plots under disease pressure and disease-free conditions were tested using an unmanned aerial vehicle (UAV) over two crop seasons. A strong relationship between grain yield, a vegetative index (MCARI2), and canopy temperature was observed under disease pressure. A strong relationship was also observed between the area under the disease progress curve of TSC and three vegetative indices (RDVI, MCARI1, and MCARI2). In addition, we demonstrated that TSC could cause up to 58% yield loss in the most susceptible maize hybrids. Our results suggest that the RS techniques tested in this study could be used for high throughput phenotyping of TSC resistance and potentially for other foliar diseases of maize. This may help reduce the cost and time required for the development of improved maize germplasm. Challenges and opportunities in the use of RS technologies for disease resistance phenotyping are discussed.

## Introduction

**Figure 1:**
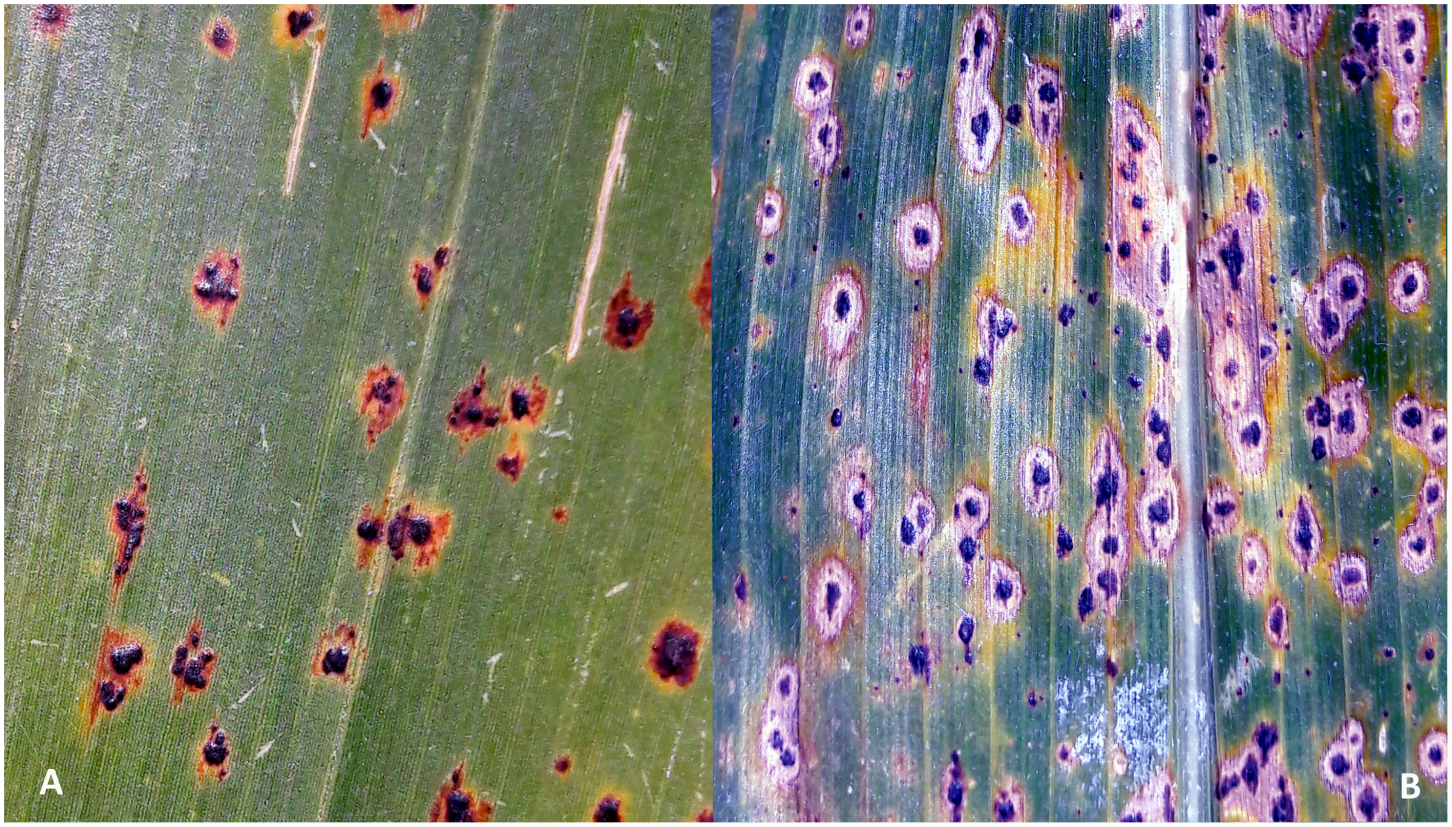
Symptoms of tar spot complex (TSC) on maize plants: **(A)** stromata of *Phyllachora maydis* appears on leaves initially; **(B)** chlorotic and necrotic spots caused by *Monographella maydis* surround the stromata of *P. maydis* ~2 weeks later causing the so called “fish-eye” symptom.

Tar spot complex (TSC) is a major foliar disease of maize in many regions of Latin America. The disease is caused by the interaction of two fungal pathogens, *Phyllachora maydis* Maubl. and *Monographella maydis* Müller & Samuels. A third fungus, *Coniothyrium phyllachorae* Maubl. is also often associated with TSC (Hock et al., 1989). The initial symptoms of the disease appear as dark oval or irregularly shaped stromata of *P. maydis* erupting through the epidermis of the lower and central leaves (Figure 1A). Approximately 2 weeks later, the area surrounding the stromata becomes chlorotic, forming a halo-like effect that is often referred to as the typical “fish-eye” symptom of TSC, which is caused by *M. maydis* (Figure 1B). Approximately within 1 month, the disease symptoms progress from lower to upper leaves. While *P. maydis* is an obligate parasite, *M. maydis* is thought to be an endophyte or facultative parasite, causing extensive chlorosis in the presence of *P. maydis* (Dittrich et al., 1991; Hock et al., 1992). It is not known whether *M. maydis* and *P. maydis* can be present as pathogens in a plant independently, or if infection in maize is triggered only by their simultaneous co-occurrence. Although *C. phyllachorae* is often isolated from the leaves infected by *P. maydis* and *M. maydis*, its role in TSC is still not clear, and it is believed to be a hyperparasite or mycoparasite, with no obvious host symptom expression (Ceballos and Deutsch, 1992; Hock et al., 1992). In the case of severe TSC epidemics, the chlorotic halos coalesce and the entire plant can become necrotic approximately within a week. Hock et al. (1989) proposed the potential involvement of phytotoxin production in TSC, as a cause of the rapid foliage “burning” effect. It was suggested that optimal temperature for the development of the disease is 16–18°C (± 5–7°C) with a monthly average rainfall of 150 mm and 10–20 foggy days per month (Hock et al., 1989).

The disease complex has been reported in several Latin American countries and was considered to be confined to the tropical areas of the region (Hock et al., 1989). Most of the information regarding yield losses caused by TSC is anecdotal. Hock (1988) mentioned losses of 30% of maize yields but did not specify the details of the study. In another study, Hock et al. (1995) reported yield losses ranging from 11 to 25% in Poza Rica, Veracruz, Mexico, although the details of this study were not revealed either. Bajet et al. (1994) reported losses of 46% in their fungicide efficacy study in the same location. To our knowledge, the latter study is the only detailed yield loss report for TSC, and it has not been updated.

Despite TSCs historical occurrence in only certain tropical areas of Latin America, *P. maydis* (alone, i.e., not in association with *M. maydis*) was detected for the first time in several locations in the Midwestern United States in 2015 (Bissonnette, 2015; Ruhl et al., 2016; Wise et al., 2016). In addition to reconfirmation at the same locations in 2016, the pathogen was also reported in Florida (Bradley, 2016; Hansen et al., 2016; Miller, 2016) and Minnesota in 2017 and 2018 (Dr. Martin Chilvers, personal communication). There have been no reports of yield losses caused by *P. maydis* alone so far; however, Mottaleb et al. (2018) have hypothesized that if TSC did form in the USA by the association of *M. maydis* with *P. maydis*, a loss of only 1% of the country-wide grain production would equate to 1.5 million metric tons worth US $231.6 million.

Thus far, phenotyping TSC resistance in maize is performed only in the field due to the absence of a reliable greenhouse inoculation technique. At the International Maize and Wheat Improvement Center (CIMMYT), disease evaluation is conducted on a 1–5 scale, in which one is very resistant (no disease symptoms) and five is very susceptible, with all foliage chlorotic and necrotic. Disease scoring is performed by visual observation every 10–14 days starting from anthesis, usually three to four times in total, depending on the disease severity and the rate of its development. Multiple previous studies, however, have reported possible inaccuracies in the visual disease ratings due to the biases of evaluators (Nutter, 1993; Newton and Hackett, 1994; Parker et al., 1995; Steddom et al., 2005; Bock et al., 2008, 2010). Inconsistencies in disease scoring may occur within the same trial both between different disease evaluators and between different scores given by the same evaluator in a given trial. Furthermore, the visual evaluations may be time-consuming and expensive, requiring highly trained and experienced technical personnel.

Remote sensing (RS) imagery has played an important role in plant phenotyping in different environments and for various crops. The objective of the technology is to minimize labor expenses, reduce the time needed for phenotyping, and to improve the accuracy of the phenotypic data. The leaf surface absorbs, transmits, or reflects light radiance differentially, depending on its internal structure, chemical composition, and plant development stage. Spectral radiometers detect electromagnetic wavelengths beyond those visible to the human eye, such as reflectance in the infrared spectrum. This information can be merged to identify specific plant features that may not be observable in the visible spectrum. Measuring spectral reflectance, therefore, can be used to understand the plant health status or to quantify the extent of the disease in afflicted parts of the plant (Simko et al., 2017). Confounding effects caused by abiotic stresses, however, must also be taken into consideration. Factors such as water and nutrient stress may also decrease the photosynthetic activity of the plant, in turn influencing leaf reflectance at the canopy level.

Laboratory-based spectroscopy has been used to detect different diseases in a number of crops (Bauriegel et al., 2011a,b; Mahlein et al., 2012, 2017; Bergsträsser et al., 2015; Kuska et al., 2015). Furthermore, low altitude field-based spectroscopy including multispectral and hyperspectral remote imagery, has been used for disease resistance phenotyping in different crops. These include powdery mildew and leaf rust in wheat; Huanglongbing disease in orange trees; root rot in cotton; *Flavescence dorée* and grapevine trunk disease in vineyard; late blight in potato; and *Xylella fastidiosa* in olive (Franke and Menz, 2007; Yang et al., 2010; Garcia-Ruiz et al., 2013; Khaled et al., 2017; Zarco-Tejada et al., 2018; Albetis et al., 2019; Franceschini et al., 2019). The majority of these studies utilized visible (VIs, mostly green and red spectrum region) and near-infrared (NIR) spectrums using hyperspectral and/or multispectral sensors. Furthermore, most of these studies used classification machine-learning algorithms for distinguishing diseased plants from non-diseased. In general, the accuracies varied from 50 to 90%, depending on the disease development stage. In some studies, red-edge spectral region, which is generally associated with canopy and leaf structural traits (e.g., leaf area index, LAI) proved to be a reliable indicator to distinguish different levels of disease severities (Garcia-Ruiz et al., 2013; Franceschini et al., 2019). Despite their successful application to various plant disease phenotyping, these RS technologies have never been used for phenotyping maize foliar diseases, including TSC.

The first objective of this study was to explore the potential of multispectral and thermal imaging using an unmanned aerial vehicle to phenotype TSC resistance in maize, and to compare the effectiveness of this method with that of conventional visual disease evaluation. The second objective was to assess potential grain yield losses to TSC in a humid lowland tropical environment in Mexico.

## Materials and Methods

### Plant Material

Twenty-five tropical and subtropical maize hybrids were selected for the experiment (Table 1). All hybrids had been previously evaluated for agronomic performance and resistance to TSC in multiple locations in Mexico (data not shown). The hybrids included two resistant (CLTHW13007 and CLTHW13008) and two susceptible (DTMA-112/DTMA-229 and DTMA-217/DTMA-207) controls, and their status was based on their reactions to the disease during previous evaluations of TSC resistance at CIMMYT (data not shown).

**Table 1:** Grain yield loss caused by tar spot complex of maize calculated as the percentage difference of grain yield (t/ha) between fungicide and non-fungicide treatments over 2016 and 2017 growing cycles at the International Maize and Wheat Improvement Center (CIMMYT), Mexico.

**Genotype entry #**	**Available pedigree or commercial name**	**Origin (company/organization)**	**Non-fungicide treatment**		**Fungicide treatment**		**% grain yield loss**
			**AUDPC^a^**	**Grain Yield (t/ha)**	**AUDPC**	**Grain Yield (t/ha)**	
1	CLTHW14001	CIMMYT	29.94	4.70	29.49	5.81	19^*^
2	CLTHW14007	CIMMYT	29.72	5.13	29.17	6.36	19^*^
3	CLTHW13001	CIMMYT	28.85	4.90	29.4	5.30	8
4	CLTHW15008	CIMMYT	49.37	4.35	29.51	4.99	13^*^
5	H-565/CML576	INIFAP	49.71	4.35	29.33	5.46	20^*^
6	P-4082W	PIONEER	92.41	3.28	29.48	4.89	33^*^
7	DK-357	DEKALB	105.58	1.91	32.46	4.19	54^*^
8	XT-3402	ASPROS	46.23	4.25	29.17	5.26	19^*^
9	ZAPATA-7	CAUDILLO	73.01	2.89	29.65	4.48	35^*^
10	REGATA	REGA	48.31	3.56	29.67	4.94	28^*^
11	Imparable	BERENTSEN	75.57	2.3	29.43	3.01	23^*^
12	PS-464	POWER	86.66	2.08	30.47	4.98	58^*^
13	CLTHW13003	CIMMYT	56.96	4.04	29.82	5.16	23^*^
14	CLTHW11001	CIMMYT	63.41	4.01	29.75	5.62	27^*^
15	CLTHW13006	CIMMYT	60.06	3.04	29.28	4.03	25^*^
16	CSTHW13003	CIMMYT	102.21	2.38	31.75	5.14	54^*^
17	CSTHW13004	CIMMYT	96.86	3.08	32.63	4.95	38^*^
18	CSTHW13005	CIMMYT	105.26	2.42	35.54	4.64	48^*^
19	CSTHW14007	CIMMYT	114.15	1.53	35.53	3.58	57^*^
20	CSTHW14008	CIMMYT	103.37	2.01	30.02	4.21	52^*^
21	CSTHW14009	CIMMYT	112.99	1.89	31.42	4.24	55^*^
22	Resistant Check 1^b^	CIMMYT	48.69	4.47	29.74	4.54	1
23	Resistant Check 2	CIMMYT	54.41	4.09	29.4	5.29	23^*^
24	Susceptible Check 1	CIMMYT	120.19	2.21	30.79	3.56	38^*^
25	Susceptible Check 2	CIMMYT	117.04	2.35	35.17	4.20	44^*^

The asterisk (

* *) indicates that a significant difference between the two treatments (i.e., fungicide vs. non-fungicide) for the genotypes was detected at p ≤ 0.05*.

a *Area under disease progress curve*.

b *Resistant Check 1, CLTHW13007; Resistant Check 2, CLTHW13008; Susceptible Check 1, DTMA-112/DTMA-229; Susceptible Check 2, DTMA-217/DTMA-207*.

### Inoculation and Disease Scoring

**Figure 2:**
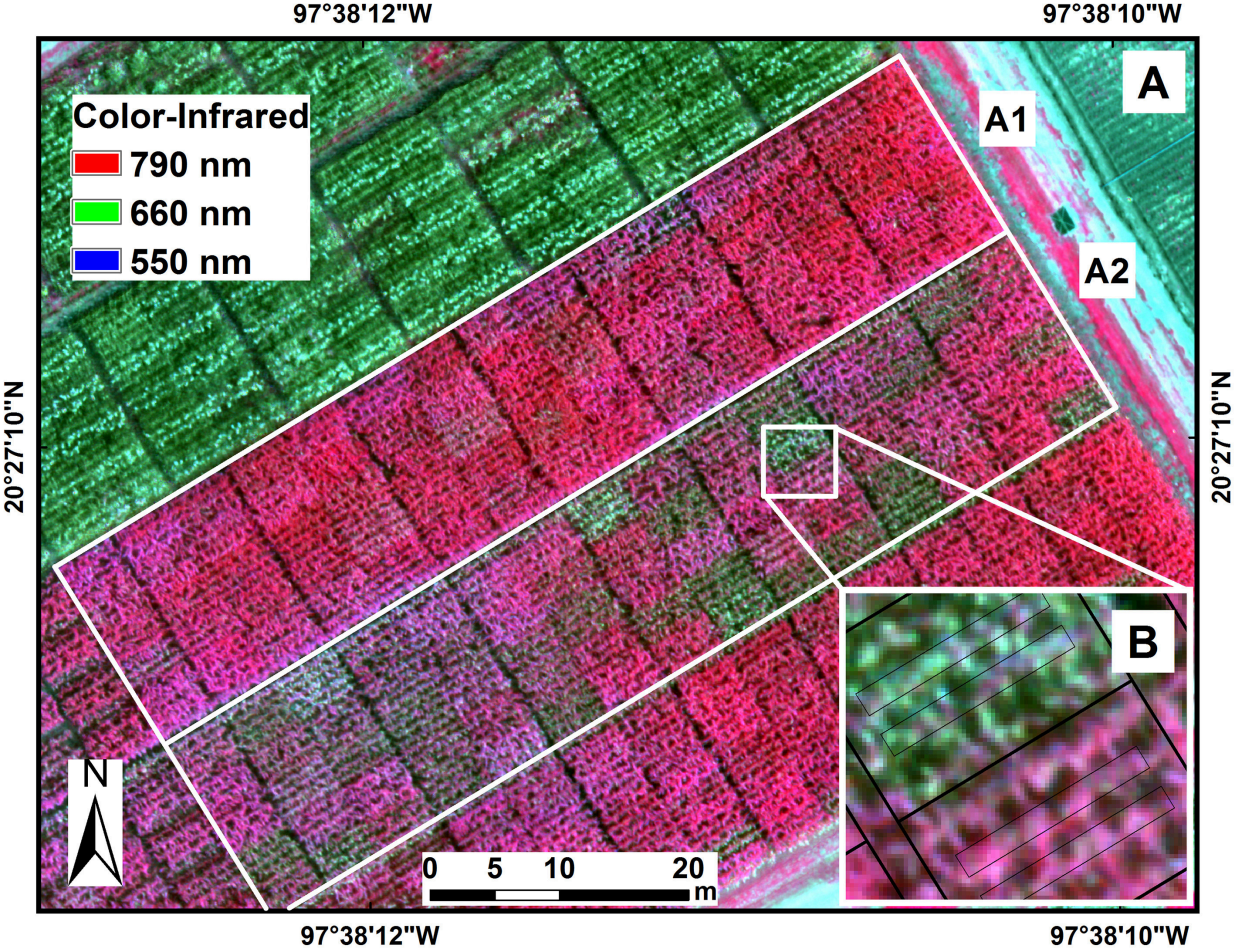
Location of the experiment at the International Maize and Wheat Improvement Center (CIMMYT), Agua Fria experimental station in the state of Puebla, Mexico **(A)**. Color-infrared image (790, 660, 550 nm) of maize hybrids in the experimental trials under fungicide treatment (A1) and non-fungicide treatment (A2) of tar spot complex of maize. Image data were extracted from two polygons from the two central rows in each plot **(B)**.

The experiment was conducted during the winter–spring growing cycles of 2016 and 2017 at CIMMYT's Agua Fria Experimental Station in the north of the state of Puebla, Mexico (20.45°N, 97.64°W) at 110 m above sea level (Figure 2). The typical annual precipitation at the station is ~1,200 mm and the air temperature ranges from 5 to 42°C during the winter growing cycle (November–April), with average relative humidity of 85%. The soils are clay loam with a pH of 7.5–8.5. Low temperatures, high relative humidity, and extended leaf wetness during this period also favor the development of Northern Leaf Blight (NLB) of maize, caused by *Setosphaeria turcica* (Luttrell) Leonard and Suggs [anamorph *Exserohilum turcicum* (Passerini) Leonard and Suggs]. To avoid coinfection with NLB and to favor the development of TSC, planting was delayed by 2 months, and crops were planted in late January. This promoted optimal development conditions for TSC while reducing the risk of NLB infection.

The inoculation was conducted as follows: a mix of several TSC-susceptible but NLB-resistant maize genotypes was planted 30–45 days prior to the experiment on a small plot (~10–20 m^2^) in the same location as the experiment. These plants were infected naturally by TSC during the late stages of plant development and provided the initial inoculum for the trial. Leaves (~100–150, in total) from these susceptible plants with fully expressed TSC symptoms were collected, submerged in water for several seconds to wet the leaf surface, and placed into a 200-l plastic barrel, closed with a lid, and left to incubate under shade for ~24 h. Then, the barrel was filled with water, the leaves were agitated lightly, and the resulting spore suspension was filtered through a coarse sieve to remove large leaf debris. Tween-20 (Sigma Aldrich, St. Louis, MO, USA) was added to the water/spore suspension (1 ml per 15 l of inoculum) as a surfactant. The spore suspension was sprayed over the maize foliage with a handheld sprayer after 6–7 pm to make use of the period of dew-induced leaf wetness during the night. The inoculation was carried out twice, with the first performed before the tasseling stage and the second 7 days later.

Disease assessments were conducted three to four times per growing season with 10-day intervals between each assessment, starting at anthesis. The disease scoring was conducted throughout plots using a 1–5 disease rating scale, in which: 1 = highly resistant or close to immune reaction with nearly 0% of leaves infected and with no visible stromata of *P. maydis*; 2 = a resistant to moderately resistant reaction with 1–25% of the leaf area affected by a few scattered stromata of *P. maydis*; 3 = a moderately resistant to moderately susceptible reaction with 26–50% of the leaf area affected with moderate densities of chlorotic lesions with well-developed “fisheye” symptoms induced by *M. maydis*; 4 = a susceptible reaction with 51–75% of the leaves affected with large coalesced chlorotic and necrotic spots; 5 = a highly susceptible reaction with 76–100% of the leaves affected with extensive necrosis and often premature senescence of the plants.

### Experimental Design

The crops were planted in two side-by-side blocks in a square lattice design, each with three randomized replicates, at the end of January 2016 and January 2017. One block was TSC-free (disease controlled by fungicide applications, herein referred to as “fungicide treatment”; Figure 2A1) and the other was TSC-infected (without fungicide control, herein referred to as “non-fungicide treatment,” Figure 2A2). The experimental plots consisted of four 5 m long rows spaced 60 cm apart, with ~20–25 cm within-row spacing between plants. Plots were fertilized with N-P-K at 150-80-30 according to the recommended doses based on the soil analysis. Irrigation was applied as required. To prevent fungicide drift between blocks, four rows of closely planted maize plants (filler rows), and a 1.5 m wide, empty strip of land were used to separate the two blocks. In the fungicide treatment block, the disease was controlled using the fungicide Priori Xtra^TM^ (Azoxystrobin 18.2 + Cyproconazole 7.27, Syngenta Crop Protection, Greensboro, NC) at a rate of 1 l/ha. The fungicide was applied with handheld sprayers at 7- to 10-day intervals at least six times during each growing cycle. Only two internal rows were harvested, and grain yield was measured in tons per hectare with the grain moisture adjusted to 12.5%.

### Remote Sensing and Data Processing

The flights were carried out using a fixed-wing UAV-based RS eBee platform (SenseFly Ltd., Cheseaux-Lausanne, Switzerland) weighing <2 kg including camera and battery. The nominal radio link range of the platform was 3 km with a maximum flight time of ~30–35 min, a cruise speed of 11–25 m/s, and resistance to winds of up to 12 m/s. The UAV was equipped with a multispectral MultiSpec 4C camera, which provided spectral images at 550 (40 nm full width at half maximum, FWHM), 660 (40 nm FWHM), 735 (10 nm FWHM), and 790 nm (40 nm FWHM), and a ThermoMAP (7.5–13.5 μm) thermal infrared camera (Airinov, Paris, France). The two cameras were mounted separately, and successive flights were conducted with different cameras. The UAV flew 55 m above the ground at midday in sunny conditions, covering an area of 1.7 ha (i.e., an area 0.2 ha larger than the experimental area, in order to obtain accurate orthomosaics). The images were acquired with 80% lateral and 90% longitudinal overlaps, flying north/south and east/west. This resulted in a ground resolution of 6 and 12 cm for the multispectral and thermal cameras, respectively. For the multispectral camera, radiometric calibrations, and corrections were performed before each flight using the standard camera panel provided by the manufacturer. In addition, during each flight, sun irradiance was measured by the incident light sensor built into the multispectral camera, allowing for radiometric adjustment of images taken under different light conditions. The flights and visual disease assessments were conducted on the same days, starting at anthesis. Four and three flights were performed in 2016 and 2017, respectively.

The images were geotagged for orthomosaic processing using Pix4D Mapper® software (v3.3.24; Pix4D, Lausanne, Switzerland). The images were converted into reflectance and surface temperature for the multispectral and radiometric thermal infrared data, respectively. In total, eight different vegetation indices (VIs) were calculated for each orthomosaic. Structural VIs were: normalized difference vegetation index (NDVI), renormalized DVI (RDVI), optimized soil-adjusted vegetation index (OSAVI), modified simple ratio (MSR), and modified chlorophyll absorption in reflectance indices (MCARI1 and MCARI2), while pigment specific simple ratio for chlorophyll A (PSSRa) was used as a chlorophyll-related index and green (G) was used as a red- green-blue (RGB) ratio index (Table 2). For each wavelength required to calculate the VIs, the closest wavelength response of the multispectral signal was considered, taking into account the FWHM of each channel. Canopy temperature was estimated using the thermal infrared signal.

**Table 2:** Relationship between grain yield of maize hybrids and area under the disease progress curve (AUDPC) of tar spot complex with areas under different wavelengths, vegetative indices and thermal imagery under fungicide and non-fungicide treatments.

**Indices**	**Equation**	**Relationship (*R*^2^) with yield**		**Relationship (*R*^2^) with AUDPC**	
	**Wavelengths**	**Fungicide treatment**	**Non-fungicide treatment**	**Fungicide treatment**	**Non-fungicide treatment**
W550 (550 nm)		0.00	0.40^*^	0.20^**^	0.50^*^
W660 (660 nm)		0.08	0.59^*^	0.02	0.72^*^
W735 (735 nm)		0.08	0.72^*^	0.28^*^	0.85^*^
W790 (790 nm)		0.37^*^	0.79^*^	0.26^*^	0.91^*^
**STRUCTURAL INDICES**					
Normalized difference vegetation index (NDVI) (Rouse et al., 1973)	$\left(\frac{R_{800}-R_{670}}{R_{800}+R_{670}}\right)$	0.28^*^	0.76^*^	0.02	0.90^*^
Renormalized DVI (RDVI) (Roujean and Breon, 1995)	$\left(\frac{R_{800}-R_{670}}{\sqrt{R_{800}+R_{670}}}\right)$	0.40^*^	0.79^*^	0.13	0.93^*^
Optimized soil-adjusted vegetation index (OSAVI) (Rondeaux et al., 1996)	$\frac{\left(1+0.16\right)*\left(R_{800}-R_{670}\right)}{{(R}_{800}+R_{670}+0.16)}$	0.35^*^	0.79^*^	0.08	0.92^*^
Modified simple ratio (MSR) (Chen, 1996)	$\frac{R_{800}}{R_{670}}-1/{\left(\frac{R_{800}}{R_{670}}\right)}^{0.5}+1$	0.03	0.55^*^	0.06	0.57^*^
Modified chlorophyll absorption in reflectance index (MCARI1) (Haboudane et al., 2004)	1.2^*^[2.5^*^(*R*_800_−*R*_670_)−1.3^*^(*R*_800_−*R*_550_)]	0.37^*^	0.79^*^	0.24^*^	0.93^*^
Modified chlorophyll absorption in reflectance index (MCARI2) (Haboudane et al., 2004)	$\frac{1.2*\left[2.5*\left(R_{800}-R_{670}\right)-1.3*\left(R_{800}-R_{550}\right)\right]}{\sqrt{{\left(2*R_{800}+1\right)}^{2}-\left(6*R_{800}-5*\sqrt{R_{680}}\right)-0.5}}$	0.36^*^	0.81^*^	0.16^**^	0.93^*^
**CHLOROPHYLL INDEX**					
Pigment specific simple ratio for chlorophyll A (PSSRa) (Blackburn, 1998)	$\left(\frac{R_{800}}{R_{680}}\right)$	0.16^**^	0.79^*^	0.00	0.87^*^
**RGB RATIO**					
Green (G) (Zarco-Tejada et al., 2005)	$\left(\frac{R_{550}}{R_{670}}\right)$	0.05	0.76^*^	0.14	0.88^*^
**THERMAL IMAGERY**					
Canopy temperature		0.36^*^	0.81^*^	0.15	0.89^*^
**VISUAL DISEASE EVALUATION**					
AUDPC (Vanderplank, 1963)		0.14	0.84^*^	–	–

* *p < 0.01*;

** *p < 0.05*.

The image data were extracted from only the two central rows (out of four rows) of each plot since only those were harvested for the grain yield estimation. Two polygons were outlined (Figure 2B), one for each central row. The area of 0.2 × 0.5 m surrounding the two central rows was considered a buffer zone. The pixels were selected and averaged from the inside of the polygons using ArcGIS® software (v10.1; ESRI, Redlands, CA, USA).

### Data Analysis

The Analysis of Variance (ANOVA) was performed to determine if the maize genotypes performed similarly between the experimental years in terms of grain yield reduction caused by TSC. The phenotypic data were analyzed with a standard linear mixed model in which the year, replication, and plots within replication were considered as random effects. The treatments (fungicide and non-fungicide) and genotypes were considered as fixed effects. The disease data obtained through visual scoring across the two cycles were summarized and analyzed using the area under the disease progress curve (AUDPC) with the trapezoidal method i.e., Riemann's integrals (Vanderplank, 1963). The area under the curve (AUC) for the individual wavelengths, vegetative indices (VIs) and thermal imagery was also obtained using Riemann's integrals. This allows integration of the temporal information from the imagery data and the disease measurements into single variables (AUDPC and AUC). Differences in individual maize genotype performance were evaluated in terms of grain yield losses (t/ha) for each entry under the two treatments. The data from AUDPC and AUCs were used for individual association by means of regression analysis with grain yield and with each other. The analyses were performed using the statistical software R, version 3.3.3, and its respective libraries for mixed models and multiple comparison procedures (Bates et al., 2015; Lenth, 2016; R Core Team, 2017).

## Results

### Effect of Tar Spot Complex on Grain Yield

The results of ANOVA indicated that the effect of interaction of experimental years with the maize genotypes was not significant (*p* > 0.05, data not shown). Therefore, the effect of TSC was analyzed across both experimental years. The disease development was optimal during both cycles with the susceptible checks reaching the highest AUDPC values among all genotypes across the 2 years (Table 1). The disease control in the fungicide treatment was also optimal with only traces of the disease observable on the lower foliage, resulting in low AUDPC scores. This explained the strong relationship between the yield and AUDPC (Table 2) in the non-fungicide treatment (*R*^2^ = 0.84). As expected, the relationship between AUDPC and grain yield in the fungicide treatment was weak (*R*^2^ = 0.14).

Comparing the performance of individual genotypes between the two treatments (fungicide vs. non-fungicide) revealed that the yields of most of the genotypes were affected (lowered) by the disease under the non-fungicide treatment (Table 1). The only exceptions were CLTHW13001 and the Resistant Check 1, with yields which were not significantly different (*p* > 0.05) between the two treatments. The highest yield loss of 58% was observed in PS-464.

### Remote Sensing

The analysis of the relationship of grain yield with the AUCs of the individual wavelengths, VIs, canopy temperature, and the AUDPC across the 2 years revealed that the majority of the RS variables were strongly correlated with yield under the non-fungicide treatment (*p* ≤ 0.01, Table 2). Under the non-fungicide treatment, the coefficient of determination (*R*^2^) of the interactions between the grain yield and the individual wavelengths (550, 660, 735, and 790 nm) was 0.40–0.79. The *R*^2^ of the relationship of the structural VIs (NDVI, RDVI, OSAVI, MSR, MCARI1, and MCARI2) was 0.55–0.81. Furthermore, the *R*^2^ of the relationship of the chlorophyll index PSSRa and RGB ratio G with grain yield was 0.79 and 0.76, respectively. These data imply that, the genotypes with higher AUCs for these variables, with the exception of W660, also had higher yields under disease pressure. In contrast, under the non-fungicide treatment, the *R*^2^ of the canopy temperature and AUDPC were 0.81 and 0.84, respectively. This indicated that the genotypes with lower canopy temperature AUC values and lower AUDPC scores had higher yields under disease pressure. While the relationships for different wavelengths, VIs, canopy temperatures, and AUDPCs with grain yields were noticeably higher under the non-fungicide treatment, these relationships were predictably weaker under the fungicide treatment. This is explained by the absence of the disease i.e., photosynthesis, and thus grain yield, were unaffected in un-diseased plants. Under the fungicide treatment, the relationship of W550, W660, W735, MSR, and G with yield was not significant (*p* > 0.05), however, W790 nm, the other indices and canopy temperature had a significant (*p* ≤ 0.01) relationship with yield. Among all RS variables, MCARI2 and canopy temperature had the strongest relationship with grain yield (*R*^2^ = 0.81 for both) under disease pressure (Table 2). Furthermore, MCARI1, MCARI2, and RDVI showed the strongest relationships with AUDPC values (*R*^2^ = 0.93 for each index).

## Discussion

Our results suggest that potential yield losses from TSC in maize hybrids may be as high as 58% in susceptible genotypes under strong disease pressure. This number is considerably higher than the 46% reported by Bajet et al. (1994). Although the effect of the disease on inbred lines was not investigated in our study, grain yield losses would also likely be extensive. The strong relationship between AUDPC and grain yield (*R*^2^ = 0.84) indicates the overall impact TSC could have on maize production following a severe epidemic in susceptible maize germplasm. The severity of TSC infection at Agua Fria Station over the 2 years during which the experiment ran was not exceptionally strong, so our results present a conservative estimate of yield loss. Natural epidemics of greater severity, which do occur occasionally, may lead to even higher grain yield losses.

The onset of disease in relation to maize growth stage (e.g., disease symptoms appearing before or after flowering) may have a major effect on yield losses, although this hypothesis requires further testing. Another factor influencing yield losses may be the role of *M. maydis* in TSC epidemiology. Hock et al. (1989) suggested that *M. maydis* may produce toxins causing accelerated senescence-like effects on maize foliage. In separate studies (Loladze et al., unpublished data), detected the presence of such phytotoxins in several isolates of *M. maydis*. The differences in phytotoxin production between the isolates possibly suggests the existence of different races within populations of *M. maydis*. Depending on the phytotoxin production properties of the races present in a particular location and year, the disease severity and extent of damage caused by TSC may vary significantly, explaining yearly fluctuations in maize yield losses. A study to test this theory is underway.

An additional indirect factor affecting yield losses caused by TSC could be ear rots, which are often found in plants weakened by TSC (Loladze et al., unpublished data). Although rots are not caused directly by the same pathogens causing TSC, they could play a significant role in overall yield loss dynamics. The interaction between TSC and ear rots, therefore, also needs to be addressed in a separate study.

The application of hyperspectral signals for phenotyping disease resistance in a number of crops has been discussed extensively by Shakoor et al. (2017) and Simko et al. (2017). Examples of such diseases include stripe rust and fusarium in wheat (Bauriegel et al., 2011a; Devadas et al., 2015), sugarcane orange rust (Apan et al., 2004), *Venturia inaequalis* in apple trees (Delalieux et al., 2007), and red leaf blotch in almond (López-López et al., 2016). All of the above-mentioned studies reported high accuracies of hyperspectral signals when applied to disease resistance phenotyping.

The results of the current study, however, demonstrate the potential use of multispectral imaging for maize disease evaluation, a method considerably less expensive than the use of hyperspectral cameras. Several previous studies demonstrated that NDVI, a structural index calculated on the basis of red, and NIR wavelengths, was moderately to highly accurate in distinguishing different levels of severity for wheat diseases and insect pests. These included leaf rust and stripe rust of wheat, and sunn pest (Franke and Menz, 2007; Genc et al., 2008; Pretorius et al., 2017). In our study, however, NDVI was not the most accurate index in terms of correlation with grain yield losses in maize caused by TSC (*R*^2^ = 0.76).

Our study showed that a number of VIs calculated from a multispectral signal and thermal data were highly correlated with disease severity and grain yield under non-fungicide treatments. The strongest relationship with yield was observed with MCARI2 VI and canopy temperature (*R*^2^ = 0.81 for each, Table 2). This, however, was weaker than the relationship between AUDPC and grain yield (*R*^2^ = 0.84). In addition, the strong relationship between MCARI2 and AUDPC (*R*^2^ = 0.93) suggests that this index could potentially serve as an auxiliary instrument for large-scale disease trials, especially on high throughput phenotyping platforms. While the relationship between grain yield and AUDPC was still slightly stronger under the non-fungicide treatment (*R*^2^ = 0.84), both canopy temperature and MCARI2 could potentially be used to assess disease resistance and possibly forecast yield losses caused by TSC in maize. Therefore, the relationship between the AUDPC and grain yield was still slightly higher than that between AUC and the grain yield. Nevertheless, RS still has a potential application to disease phenotyping on large-scale high throughput phenotyping platforms. In such cases, the visual scoring would be excessively laborious time consuming.

VI MCARI2 is an improved version of MCARI, which was modified in order to reduce the noise effects of soil within the reflectance signal while preserving the sensitivity to canopy leaf area index (LAI) and resistance to chlorophyll content variability (Haboudane et al., 2004). Previously, a spectrum region associated with canopy and leaf structures (red-edge) was reported to be sensitive enough to differentiate various levels of disease severity (Garcia-Ruiz et al., 2013; Franceschini et al., 2019). Similar results were found in our study where red-edge and NIR (735 and 790 nm) showed strong relationships with yield and AUDPC under non-fungicide treatments (Table 2). However, those wavelengths had still slightly lower relationship with yield and AUDPC as compared with MCARI2.

Vegetation presents two peaks of light absorption in the blue and red spectrum regions due to Chlorophyll content (C_a+b_), high reflectance in green, while its biomass and canopy structure are related to reflectance in red-edge and NIR spectrum regions (Richardson et al., 2002). The VIs are used for combining multispectral observations into single metrics, which minimize the effect of external factors on spectral data and derive specific canopy characteristics (Baret and Guyot, 1991). These facts and a possible reduction of LAI caused by the TSC-induced decrease of photosynthetic activity, led us to consider that MCARI2 could also be used as a potential alternative for assessment of yield losses caused by the disease.

In some cases, plant disease development may be associated with temperature changes in the foliage of the diseased plants, which can be measured by infrared thermography (Simko et al., 2017). Some plant diseases may induce stomatal closure, thus reducing evaporative cooling and increasing canopy temperature (Chaerle et al., 2001; Calderón et al., 2015). In the current study, although notable differences in canopy temperatures between resistant and susceptible genotypes were observed, no progressive curve of canopy temperature paralleling the disease development curve was detected (data not shown). Nevertheless, when the canopy temperature data were used to calculate the AUC, such time-series information showed a strong relationship with the yield data under the non-fungicide treatment (*R*^2^ = 0.81). The absence of a growth curve for the canopy temperature could have been influenced by the field conditions (e.g., ambient temperatures, thermal radiation, or sunlight intensity) during the flights and/or by the stage of plant development and its interaction with the pathogen (Chaerle et al., 1999, 2004; Lindenthal et al., 2005; Simko et al., 2017). At the same time, it is noteworthy that our experimental plots were not subjected to water stress or nutrient deficiencies, factors that could have influenced evapotranspiration, and thus canopy temperature.

Provided that abiotic stresses, such as drought and soil nutrient deficiencies, are minimized during disease trials, RS can play an important role in high throughput phenotyping for resistance to foliar diseases of maize. This can potentially decrease the likelihood of human error and reduce the workload of visual scoring on a large scale (Mahlein, 2016). With ongoing improvements in RS technology and image data analysis techniques and procedures, the relationships between plant traits and imaging data may be further enhanced. This may lead to wider and more common application of RS technology in maize breeding on large-scale and multi-location phenotyping platforms.

While RS technology has potential to significantly innovate high throughput phenotyping of disease resistance, barriers limiting the introduction of the technology into breeding programs remain. Large initial investments for purchasing the system, highly trained specialists for image acquisition and processing, and potential delays in data processing are some of the challenges (Khaled et al., 2017). Although RS technologies, which include cameras, platforms, and data processing software are becoming more affordable, systems and protocols need to be adapted to the phenotyping requirements of particular, economically important maize diseases.

The use of RS for early disease impact evaluation and the detection of interactions of biotic and abiotic stresses requires further investigation, as also suggested by previous authors (Calderón et al., 2013; Mahlein, 2016; Khaled et al., 2017; Simko et al., 2017). With the constant advancement of the RS technology, the possibilities of pre-symptomatic early detection of plant diseases, as this was recently done in olive crops, still remains to be explored in maize (Zarco-Tejada et al., 2018). This could potentially help target maize diseases with appropriate disease management interventions before the development of severe epidemics.

## Author Contributions

AL designed and conducted the field study and was responsible for the plant pathology component. FR designed and conducted the Remote Sensing component of the study. AL and FR contributed to the project equally. FT conducted the statistical analysis. FS provided germplasm and contributed to the manuscript. BG and MB provided critical insights into the manuscript writing.

### Conflict of Interest Statement

The authors declare that the research was conducted in the absence of any commercial or financial relationships that could be construed as a potential conflict of interest.
